# Understanding the needs and experiences of young cancer patients, caregivers and healthcare professionals in the UK following childhood fertility tissue preservation (FTP): a qualitative study informed by patient and public involvement and engagement

**DOI:** 10.1136/bmjopen-2024-088025

**Published:** 2025-07-28

**Authors:** Rebecca Mottram, Richard Feltbower, Georgina Louise Jones, Sarah Gelcich, Katherine McLean, Catherine Kelly, Adam Glaser

**Affiliations:** 1 University of Leeds, Leeds, UK; 2 Leeds Beckett University, Leeds, UK; 3 PPIE Representative, Leeds, UK

**Keywords:** Subfertility, Patient Participation, Paediatric oncology, SEXUAL MEDICINE, Follow-Up Studies

## Abstract

**Abstract:**

**Background:**

Childhood cancer treatment can cause subfertility in adulthood. Ovarian or testicular tissue preservation is a rapidly evolving field with significant potential benefits. However, the establishment of patient-centred reproductive survivorship pathways remains a challenge in clinical settings due to a lack of robust evidence to inform its development. Patient and public involvement and engagement (PPIE) consultation may help ensure that future studies align with patient needs and that tailored survivorship care pathways are developed for young people with preserved fertility tissue.

**Aim:**

This PPIE consultation aimed to identify priority areas for future research that would support the development of a tailored survivorship care pathway for childhood cancer survivors who have preserved tissue for future fertility.

**Methods:**

Recruitment occurred through national networks, including collaborations with advocacy groups such as Candlelighters and clinical networks. Data were collected via telephone or online unstructured interviews, with some supplementary email exchanges. Thematic analysis was used to identify emergent themes. The Guidance for Reporting Involvement of Patients and the Public (GRIPP)-2 guidelines were used to help guide PPIE.

**Setting:**

An online focus group and/or a one-to-one interview with e-mail interactions.

**Participants:**

In total, 12 unique participants took part in a focus group and/or interview. Participants included parents of children who had stored tissue, young adult cancer survivors with stored tissue and five clinicians from the leading National Health Service (NHS) centres in the UK.

**Results:**

Six key themes emerged that highlighted unmet needs and priority areas for research: (1) Lack of communication and information; (2) unmet needs in follow-up care; (3) emotional impact and psychological support; (4) importance of patient and parental involvement; (5) desire for information and education; and (6) long-term concerns and support. Parents, young adults and healthcare clinicians found talking about fertility issues difficult. They noted that consistency of care, education resources and access to emotional support were important areas where improvements could be made. We used thematic analysis to help identify patterns in the data, and we used the Guidance for Reporting Involvement of Patients and the Public (GRIPP)-2 reporting guidelines for PPIE work.

**Conclusions:**

PPIE provided valuable insights into the experiences of childhood cancer survivors with preserved fertility tissue, their parents and clinicians, highlighting priority areas to guide future research and ensure it addresses the concerns of care recipients. Our findings suggest that childhood cancer survivors who preserve tissue for future fertility need personalised follow-up care with information and psychological support. A larger sample of participants, studied using a qualitative research design, is needed to capture the full range of experiences, needs and preferences and to ensure that care is inclusive and relevant to the wider population.

**STRENGTHS AND LIMITATIONS OF THIS STUDY**

- Recruitment through national charities, clinical networks and data organisations facilitated access to a diverse group of participants.
- The use of informal, one-to-one communication techniques with participants encouraged patient and public involvement and engagement participants to share candid and detailed responses.
- The small sample size limited the generalisability of the findings, and contextual factors influencing participants’ experiences were not fully explored, as the objective was to prioritise practical contributions over a detailed contextual analysis.

Article Summary

This study used Patient and Public Involvement and Engagement (PPIE) consultation to identify priority areas for future research. Parents, young adults and clinicians identified that a lack of communication, emotional support and educational resources was all negatively impacting the quality of survivorship care. There is an urgent need for research that aims to address these gaps and better understand and meet the survivorship needs of individuals who have undergone fertility tissue preservation, ensuring comprehensive care, accurate information and appropriate support.

## Introduction

Survival rates for patients with childhood cancer have greatly improved, with more than 80% surviving at least 10 years after their initial treatment.^1^ However, many of them face long-term challenges in their health, emotions and social lives, with subfertility being a common concern.^2^,^4^ Fertility tissue preservation (FTP), involving surgical removal and freezing of ovarian or testicular tissue, offers the potential for restoration of fertility after treatment, particularly for prepubescent children at high risk of fertility issues.^5^,^7^ Between 2012 and 2019, a fourfold increase in FTP procedures established it as a standard component of paediatric cancer care.^8 9^

While FTP has advanced over decades, its success in restoring fertility is limited, with only 200 live births globally.^10^ FTP has significant implications for clinical care and resource allocation, in particular, patient counselling and long-term management of stored tissue psychosocial care of patients.^11 12^ Cancer survivors who undergo FTP are at increased risk of suffering unmet needs in survivorship and require specialist care.^13^,^20^ However, there is no established consensus on best practice for reproductive care for childhood cancer survivors with stored tissue.

Healthcare policy and practice must align with the expectations and priorities of care recipients, ensuring care is both patient-centred and relevant to their needs.^21^ Patients increasingly seek active involvement in decisions about the design of their treatment and care.^22^,^24^ These principles are particularly crucial in areas of care where standardised care pathways have not yet been established, and evidence to inform clinical practice is limited.^25 26^ Research consultations with adolescents and young adults have proven effective in designing care that is tailored to their unique needs.^27 28^

A scoping review of young adult cancer survivors’ experiences and preferences in reproductive survivorship care after FTP revealed a critical gap in the literature, particularly in understanding patient-reported outcomes and lived experiences.^29^ The 2024, James Lind Alliance Female Fertility Preservation Priority Setting Partnership established a critical step toward being able to identify and prioritise the most important unanswered questions in female fertility preservation based on the perspectives of patients, carers and clinicians.^30^ However, it may have limited capacity to generate targeted insights that specifically address the unique needs and challenges associated with FTP.

Public involvement in research involves active partnership between patients, carers and members of the public with researchers, influencing research priorities and ensuring its relevance.^31^ This approach is increasingly recognised as an essential component of patient-centred care, shaping and clarifying research priorities as well as ensuring the translation of research into clinical practice is transparent, inclusive and accessible. By actively engaging patients and other stakeholders, PPIE provides critical insights that can help bridge the gap between research and the development of tailored care pathways.^31 32^

This PPIE consultation aimed to identify key areas for future research that could be used to guide the development of a tailored survivorship care pathway for childhood cancer survivors who have preserved tissue for future fertility.

## Methods

This study used a consultative PPIE approach, following INVOLVE^33^ guidance, to seek feedback, experiences and opinions from public contributors in order to inform decision-making and identify research priorities.^31 34^

### Recruitment

Participants were recruited through a combination of professional networks, advocacy groups and direct outreach efforts between September 2024 and January 2024. Collaborators included useMYdata, DATACAN, Candlelighters, Children’s Cancer and Leukaemia Group and Teenage Cancer Trust. E-mail invitations were disseminated through collaborators' websites and email networks. The lead researcher (RM) presented the project and participation opportunity at the Leeds Young Owls and the Leeds Children’s Research Forum. RM contacted clinicians in the UK clinical network of colleagues caring for children and young people who store ovarian or testicular tissue in the UK to seek direct engagement. A poster outlining the project and participation details was designed with input from PPIE research partners and shared across collaborator platforms.

### Participants

In total, 12 unique participants took part in a focus group and/or interview. Six adult participants took part in a focus group, two of whom also participated in individual interviews. Additionally, four clinicians who had not participated in the focus group were interviewed.

### Participation format

A focus group followed by informal, one-to-one, semistructured interviews with individuals was conducted by RM via an online meeting platform or telephone call. Additional supportive information was provided via email. The focus group lasted 1.5 hours and interviews lasted up to 1 hour. Participants were remunerated for their time according to the NIHR policy on public contributor payment. Participants were remunerated for their time according to the NIHR policy on public contributor payment.^35^

Informed consent was sought from all participants for participation via email and confirmed verbally at the beginning of each contact. Written notes were taken, including verbatim quotes, which were then transcribed by RM. Interviews were not audio recorded to maintain an informal approach, reduce potential power imbalances and help participants feel comfortable sharing personal thoughts. As this was the first contact with participants, prioritising the development of mutual trust and fostering a sense of safety and openness was essential.

Due to the sensitive nature of the topic, a one-to-one meeting format was used to create a safe space for participants to share their personal views and experiences. Open-ended questions provided a flexible framework to encourage participants to express their thoughts and opinions in a conversational manner, with follow-up questions used to encourage participants to elaborate on specific aspects of their experiences, addressing areas of interest or seeking clarification.

A full interview guide is provided in online supplemental file 1.

### Data analysis

As PPIE consultation was the methodology for this study, strict adherence to formal qualitative traditions was not essential. However, a thematic analysis based on Braun and Clarke’s approach was used to provide a flexible yet systematic process for identifying, analysing and interpreting patterns in the data.^36^ Transcripts were reviewed to achieve familiarisation with the data. Keywords and recurring content or context were highlighted and extracted manually. Coding using a post-it system was used to organise and visually categorise the data.

Manual transcription and coding of the material were chosen to capture subtleties of speech such as pauses, intonations or emotion and allow for flexibility in adjusting the coding strategy as new insights emerged. This approach enabled the researcher to focus on the individual nuances of each participant’s perspective, without the need for the infrastructure and resources required by software-based analysis. The data was then organised into groups, providing a visual overview of the information. Emerging themes were identified and assigned descriptive names.

Following initial classification, a second review of the transcripts was performed to ensure that all remaining content was allocated to one of the identified themes. Additional assessment was undertaken to detect any potentially new themes or categories that might have emerged during the analysis. Relationships between the themes were then considered, particularly how they were related to the study’s aim.

### Patient and public involvement

This piece of work reports on PPIE work and includes two PPIE participants as authors. Patients or the public were involved in the design, or conduct, or reporting, or dissemination plans of our research.

## Results

Twelve unique participants contributed to the study. Six adult participants took part in a focus group, three who were childhood cancer survivors with stored fertility tissue, two who were parents of pre-adolescent children who had stored ovarian or testicular tissue in early childhood and one who was a clinician.

Six participants were interviewed, including a young adult, a parent and four clinicians. The young adult had concerns about her fertility following treatment for childhood leukaemia. At the time of her treatment, she had not been informed about fertility preservation options, and it wasn’t until she was in her mid-20s that she learnt the extent of the impact on her fertility. The parent had a prepubescent child, who underwent treatment for a brain tumour at age three and had preserved testicular tissue prior to treatment. The four clinicians interviewed, who had not participated in the focus group, comprised a surgeon, a children’s cancer doctor, a children’s nurse specialist and two professors specialising in young people’s cancer reproductive health and FTP, working within leading National Health Service (NHS) centres in the UK. Participants were anonymised using labels (eg, P1 and P2), with P1 referring to Participant 1 (quotations):

- P1: Parent.
- P2: Young Adult.
- P3: Surgeon.
- P4: Professor of Paediatrics.
- P5: Children’s Cancer Doctor.
- P6: Children’s Nurse Specialist.

Thematic analysis of the data identified a number of interconnected themes that shed light on the complexities of FTP and survivorship care and provided valuable insights to inform the direction, priorities and design of future research towards developing a tailored survivorship pathway.

We used the GRIPP 2 reporting guidelines to ensure comprehensive and transparent reporting of the Patient and Public Involvement and Engagement (PPIE) processes.^37^

### Themes

1. Lack of communication and information: Clinicians, parents and young people expressed an urgent need for tailored information resources to help navigate discussions about FTP, including the timing of discussions.
2. Unmet needs in follow-up care: Patients, parents and clinicians identified several areas of unmet need, encompassing information, education and psychosocial support, from the time of FTP and throughout survivorship. Clinicians reported experiencing conflicting perspectives between oncologists and reproductive health specialists.
3. Emotional impact and psychological support: Patients and parents reported that FTP caused significant emotional impact. Their needs changed and evolved over time, and they described experiencing long-term burden, isolation and anxiety. Patients and parents wanted tailored emotional support to navigate these challenges effectively.
4. Importance of patient and parental involvement: Clinicians wanted to find ways to ensure they could address the differing needs of patients and parents.
5. Desire for information and education: Parents and clinicians both identified the need to provide early support to reduce the potential for decision regret. Clinicians noted that teenagers often seem uncomfortable initiating discussions about reproductive health.
6. Long-term concerns and support: Uncertainty was a recurring concern for parents, who felt unsupported in managing the emotional burden of navigating FTP during survivorship.

#### Lack of communication and information

The need for communication between patients, parents and the clinical team was a recurring theme in both the focus group and interviews. At the point of FTP, there was a preference for written information due to the overwhelming volume of information about the planned treatment, when it’s ‘really impossible to take on more information’ (Parent, focus group). However, once treatment had finished, and in the interval between treatment and considering use of stored tissue, all participants agreed that talking face to face was the best way to communicate (focus group).

Parents and clinicians expressed a desire for guidance and resources to help them broach the topic of FTP with young people, particularly in relation to the social or emotional complexities it may present. One caregiver expressed their struggle:

We've never really explained to (our son) what’s happened to his fertility. […] I mean, we've just done it without him knowing and he still doesn't know his fertility has been affected […] it’s just something we don't know how to broach or discuss with him. - Parent (P1).

As FTP happens prior to cancer treatment, usually in early childhood, there is often an interval of over a decade before it is appropriate to consider using the tissue for reproductive purposes. During this time, priorities naturally shift, especially during the transition into young adulthood as patients gain a deeper understanding of their fertility. Starting the conversation seemed to be one of the biggest challenges for parents and clinicians. One explained:

Parents say they don't know how to talk about it, they are worried about talking about it and telling their child, so they avoid the subject. Others are more confident and talk about it from the beginning, but most haven't started the conversation yet, and some need a lot of help. Some will never start that conversation. -Clinician (P6).

The topic itself seemed to present a challenge that could result in missed opportunities for education or information-giving that could help alleviate uncertainty. However, without a structure to navigate these conversations, both patients and clinicians may lack confidence to initiate discussions. A parent described:

At the time of tissue preservation, the decision was urgent. I don't think there has been anything since, and it’s been over 3 years now. I have a letter and a consent form about what they did and why. […] At the time of FTP, I couldn't have taken much more in anyway. In hindsight now, I feel that someone to check in every 12 months or so would be good. It could be 20 years, and I just feel all I've got is a letter in my safe. Someone to touch base, ask if things are still the same, offer reassurance. - Parent (P1).

Long-term follow-up appointments may provide an opportunity to support parents in navigating these conversations if oncofertility care is incorporated. However, this may depend on establishing a shared expectation, as the impact of cancer treatment can affect patients’ or parents’ ability to recall decisions made prior to treatment. A clinician told us:

Often when patients come back to the clinic, they remember nothing, so it’s difficult to know when to discuss it, and we need to help them prepare for that. - Clinician (P4).

Conversely, some parents both recalled the decision to store tissue and implied they felt a burden of responsibility:

It’s going to be our responsibility for 20 years, and I don't know anything more than that. I don't know how to bring it up or talk with (my child) about it, how do I find out? -Parent (P1).

A lack of clear direction or information about their child’s fertility status or options for using the stored tissue, combined with a decade or longer interval between tissue preservation and potential use, could leave parents feeling uncertain, disconnected and stuck in a state of limbo. One clinician commented that “*there is a significant amount of misinformation that needs to be addressed” (P4). A*nother described a sense of uncertainty that peaked during the transition from adolescence to young adulthood:

Young people don't know the information, they don't always know they have preserved fertility (tissue). It’s such a confusing and difficult thing; they want information sooner, they want to know about the future. - Clinician (P6).

Clinicians expressed challenges in providing patients with information to assist them in making informed decisions about their stored tissue. One attributed this to the rapid pace of technological advancements that have enabled the introduction of a relatively new technology into NHS care, outpacing the development of corresponding support structures needed to address its long-term effects:

Nobody envisaged this when the programme started. People were not thinking about the discussion you are talking about; they probably had something, but there is no standardisation. - Clinician (P3).

In focus group discussion, parents and patients expressed the desire for information that was *‘tailored to* (their) *unique situation’* (Young Adult, focus group) and felt that generic information would not suffice to help them navigate choices. They wanted a specialist who they could *‘talk to directly who would know about this stuff*‘ (Young Adult, focus group).

#### Unmet needs during follow-up

A lack of information and support expressed by parents as well as clinicians led to the acknowledgement of unmet needs in the survivorship period. Alongside a lack of information, clinicians felt that both patients and parents needed emotional, physical and psychosocial support from the time of FTP and throughout survivorship. One commented:

There is nothing at all to help them 5 years or more post-treatment. At the moment, we provide reassurance, talk to parents, and then send them on to the tissue storage centre. The preservation happened, but they know nothing after that. *-* Clinician (P6).

This again highlights the significance of the long interval between FTP and considering tissue use, and the lack of a structured or standardised follow-up pathway. Another clinician confirmed that they had recently seen a young adult who had not had any follow-up for 5 years. This may indicate a significant risk of late detection of issues and missed opportunities to develop preventative measures to address unmet needs.

Another clinician described the current state of follow-up care after FTP as a ‘*black hole of uncertainty’* (P5), with insufficient resources available. Such views present a strong case for research to understand and comprehensively assess needs:

How we collect follow-up data and how we meet ongoing needs is very important. What is useful for the different groups will need to be considered […]. It’s hard to gauge whether it’s the right moment to explore the topic with them [reproductive health, sexual health, fertility] so it needs to be within the context of a long-term follow-up appointment, so whatever local support can be channelled to them. - Clinician (P5).

Clinicians expressed the need to draw on insights gained by data on long-term outcomes to be able to provide evidence-based care, in particular those reported by service users. The young adult confirmed the need for sensitivity in discussions about fertility and reproductive health:

It’s really embarrassing to be asked about sexual relationships, etc., even though my mum and I are close. It can be difficult to speak to your parents about that. You don't want to upset your parents as well, so you don't say things. - Young Adult (P2).

This sense of embarrassment may be compounded by a discrepancy between the information young people require and what they feel comfortable discussing in front of their parents. Comments from clinicians suggested that they too struggled to know when to initiate sensitive discussions, particularly when translating clinical results about fertility potential:

Oncologists may perform clinical assessments of fertility, but it doesn't necessarily indicate true fertility potential. This often leads to distress among patients when they receive such information - Clinician (P4).

Considering the impact of witnessing patients in distress prompted the clinician to acknowledge there was sometimes conflict between the perspectives of oncologists and reproductive health consultants which required resolution:

It would be beneficial to reduce conflicts between the perspectives of oncologists and reproductive health consultants to ensure more consistent and comprehensive care for patients. - Clinician (P4).

There was a sense of frustration that services were not more integrated, and that this led to unmet needs among parents, patients and clinicians. In particular, clinicians seemed to want definitive answers to alleviate their uncertainties, such as research-informed guidelines to inform care decisions and follow-up care as well as provide further information to families. One stated:

Families are keen to get research findings about FTP, we have an incredible legacy, and the onus is on us to make it as robust as possible. - Clinician (P3).

They further acknowledged the importance of research with patients and parents to develop a care pathway after FTP, adding that, “*(future research) is going to impact an awful lot of people. It will benefit thousands of children*” (P3). Support for further research was echoed by the parents we spoke to, whether they had chosen fertility preservation or not, who stated that “(*research) would be welcomed by anyone as a parent, those who have chosen to do it (FTP) and those who haven't*.” - *Parent* P1).

#### Emotional impact and psychological support

Comments from clinicians indicated that they recognised the significant emotional impact of FTP on patients and their families, with one explaining:

There are a lot of worries, eg, having conversations about their fertility. Cancer survivors often have anxiety, they are depressed or suicidal etc. We must care for them in the context that we can pick up these issues, they are a very vulnerable group. - Clinician (P4).

Clinicians frequently observed their patients and families were facing complex emotional challenges and understood the impact this could have on relationships as well as how the need changes as the young person grows up.

The time of greatest need is the teenage years. As they get older, they become more empowered, but families are all different. They give consent but then their relationship with the young person can fracture, they don’t even know. Somehow, we need to help them at that age. - Clinician (P5).

Both clinicians and parents' comments indicated they carry a profound sense of responsibility in supporting a young person through the vulnerable period of adolescence to young adulthood. Parents felt an overwhelming duty to protect their child’s future, including their fertility and emotional well-being, yet they struggled with uncertainty and a lack of guidance. Similarly, clinicians recognised their responsibility to address both medical and emotional and psychosocial needs of both the patient and their family.

Clinicians acknowledged there was often a tension between wanting to make sure that even ‘sensitive’ or ‘difficult’ conversations about reproductive care were initiated proactively, but they felt conflicted about how to avoid causing an invasion of privacy (P5). Young adults seemed to value direct communication as a way to mitigate conversations about sensitive topics such as test results, as it allowed them to ask questions and receive personalised explanations in real time:

A friend told me she had just been told (by her doctor) that she can't have kids, and she is absolutely devastated. The discussion wasn't face to face… I think it’s got to be face to face, and someone else present to support them. - Young Adult (P2).

This comment described a missed opportunity for direct communication that may reflect systematic barriers towards the provision of oncofertility care. A reliance on remote or written communication may be practical in some circumstances but may fail to address the preferences of patients as well as clinicians in being able to address the complex emotional and psychosocial support needs during survivorship. The Young Adult described her own, similar experience that led to feelings of isolation from her peers:

There wasn't any support. I had to go private to see a psychologist, which helped me. Having someone who understands. Our peers have a normal life and can't understand; they can't imagine it. You need a space to speak and let it out; a support group would be good, but you need them for different stages. - Young Adult (P2).

Although the NHS may currently lack capacity to provide specialist resources, this comment suggests that accessing private services is a potential barrier to the essential emotional support they require.

The emotional impact of FTP seemed to weigh heavily on parents who experienced uncertainties and anxieties. One parent told us:

There is a long time between the procedure and him needing it or finding out he really needs it, to have nothing in between for 5–10 years, 20–30 years of nothing, just doesn't sit well. And what if parents aren't around? - Parent (P1).

All the parents and patients we spoke to expressed a need for emotional support that was tailored to the unique circumstance of having made the decision to store fertility tissue. Parents understood this was crucial, despite the inherent tension surrounding discussions about fertility and sexual health between them and their teenager or young adult. The Young Adult showed an acute awareness of their parents' challenges, presenting a shift in dynamics that could have a positive or negative effect on their relationship:

If you were someone who was infertile, it would be useful for parents to have support as it’s a huge loss for them too. My mum was really worried and upset. There wasn't any support like counseling or anything like that; it was just ‘get on with it yourself and see you next year. - Young Adult (P2).

This comment suggests young people may perceive a lack of support from clinicians as a missed opportunity for the situation to help them develop strength in their close relationships. Instead, it represented a source of anxiety for the young person, who perceived her parents as struggling and felt unable to balance these needs against their own.

#### Importance of patient and parental involvement

Clinicians' comments emphasised the importance of adopting a patient-centred approach, understanding individual preferences and providing patients with meaningful, relevant information:

We need to know what patients want and how they want to be given information. Patients often ask me how they can know what’s happening. What we are doing now is going to be different in 5 years' time, with new ways to give information. And patients often ask what is happening and how they can find out about it. - Clinician (P4).

This suggests that clinicians may be frequently asked for information they feel exists in a constantly evolving landscape, with advances in technology presenting a challenge for keeping patients informed. This presented a dilemma between the desire to integrate discussions about reproductive health after FTP as a routine part of comprehensive cancer care and the ability to provide up-to-date information. However, they acknowledged that providing regular opportunities for patients and parents to ask questions at the appropriate time was vital:

Teenagers require information about their situation because, to them, fertility tissue preservation was seen as a backup plan. They also express a need for information regarding sexual health. Parents typically have the perspective of wanting what’s best for their children and often seek additional information. - Clinician (P4).

Teenagers and parents may have distinct but interconnected needs in survivorship. While teenagers perceive FTP as a ‘back-up plan’ rather than an immediate priority, they still require information about their situation and how FTP may affect their sexual health. In contrast, parents were observed to adopt a proactive role, seeking information and guidance to safeguard their child’s future options and well-being.

The comments of clinicians indicated that they recognised the different information needs of young people and parents and were keen to use these to help shape communication strategies and support informed decision-making. They understood the differences between parents and young people when it came to their involvement:

(parents) tend to take the lead in driving discussions, while the young person may be less concerned. Parents want guidance on how to approach conversations with their child and manage the uncertainty, especially if their child is now in good health apart from fertility concerns. - Clinician (P4).

Comments from clinicians implied that although parents may take the initiative in discussing reproductive health and FTP matters, clinicians were keen to support young people to participate in these conversations. They wanted to help parents manage their feelings of uncertainty between FTP and tissue use, while recognising that parents needed to balance their own protective instincts with their child’s evolving independence.

#### Desire for information and education

Comments from the Young Adult and clinician participants revealed the possibility of significant gaps in communication and care after FTP. Both expressed a desire for information and education, and while teenagers may feel less inclined to discuss their concerns directly, they still wanted information about their situation. A clinician explained:

Teenagers require information about their situation… they want information sooner, they want to know about the future. - Clinician (P5).

Teenagers may not only be seeking information on their fertility status and clarity about the long-term implications of FTP and its connection to their broader health or life goals. Their comments suggest there is a need for timely information, regular monitoring and communication with patients may fail to address patients' concerns. The Young Adult told us about the barriers she had experienced in trying to get more information on her fertility status:

I had to push each year for blood tests; otherwise, one would be done, or only every 3–5 years, which didn't feel sufficient for me. - Young Adult (P2).

Such gaps in monitoring may leave some patients feeling frustrated or burdened by the need to repeatedly self-advocate to avoid needs being overlooked. It may suggest a systemic issue with young people not being empowered to obtain the information they want and feel they need, perhaps leaving patients who might be lacking resources vulnerable to experiencing unmet needs. Comments from clinicians acknowledged this as a potential problem:

…at the moment, (we are) not equipping patients to make the right decisions… they might regret their decision, unless we have a really clear framework for how we are going to do it - Clinician (P3).

The potential emotional and psychological impacts of decision regret on patients can be significant, with clinicians appearing keen to mitigate this risk. The young adult interviewed emphasised the importance of early discussions and support regarding fertility preservation. They disclosed that:

It was never discussed until I brought it up […] I worried about missing the late effects of cancer treatment on fertility. I also worry about other patients and young people who might not ask. I think it should be brought up sooner, e.g., as teenagers, sooner rather than later […] “I had to push hard to talk to anyone about my fertility.- Young Adult (P2).

Together, these narratives suggest that adolescent and young adult patients may appreciate the opportunity for early discussion, although the responsibility for broaching this topic may reside with clinicians. Patients, especially teenagers, may not always feel comfortable or empowered to initiate these discussions themselves.

#### Long-term concerns and support

All the parents we spoke to shared a common sentiment: FTP felt like a decision rooted in the past with little ongoing engagement since that initial moment. Many described feelings of disconnection and uncertainty about what FTP meant for their child’s future. Their experiences reflect the emotional complexity of FTP and the challenges of maintaining hope while navigating treatment and uncertainty:

After FTP, I felt there was a confidence he would reach adulthood. We were completely naive, I clung onto anything that thought he had a fighting chance. It gave us hope. But I don't know how to discuss it or chat about it. The oncologist was amazing, but FTP was not the priority at the time. Since then, there has been nothing instigated or talked about in-depth about the FTP. We hope research has advanced; we know it’s still very much to safeguard him in the future - Parent (P1).

For this parent, FTP had initially offered hope and confidence, but as time went on, they were unsure of how to understand its longer-term significance. NHS care often focuses on immediate medical needs, but survivorship care should extend beyond that, acknowledging the evolving emotional needs of patients as they progress through different phases of their survivorship. The parent comments highlighted a potential deficit in ongoing support against the competing priorities associated with cancer treatment:

We've had these chats, but always there are more pressing issues. The focus is still on scans, treatment, etc. I'm not even sure what questions I would ask.” - Parent (P1).

As time went by, FTP seemed to have faded into the background, with little proactive follow-up or opportunity for in-depth discussion about its significance. This may reflect a broader gap in survivorship care, where FTP remains secondary to cancer treatment and underexplored in the following period. The parent described the impact of this:

We've tried to be open about things related to his illness, treatment, and side effects. I don't want him to get to adulthood, and I have to tell him then. Having those conversations with other parents would be incredibly powerful and useful for me, to understand their thoughts and feelings, different perspectives.” - Parent (P1)

While they wanted to be ‘open about the illness and its after-effects, there was a sense of pressure about when to discuss it. The parent identified an unmet need of being able to connect to other parents who may have had similar experiences, accompanied by a sense of isolation and highlighting the need for supportive networks and opportunities for discussion with the wider care team. Finally, there was a lingering sense of fear about losing vital information:

I still feel like if I lose this bit of paper, I've only got that. - Parent (P1)

This suggests the fragility of their connection to the FTP process, highlighting the need for more consistent communication and reassurance from the clinical team to help families feel supported, informed and able to fulfil their sense of needing to safeguard their child’s future.

## Discussion

This study gathered insights from twelve PPIE participants, including childhood cancer survivors, parents and healthcare professionals specialising in FTP. Analysis revealed six key themes that highlighted unmet needs and priority areas for research: (1) lack of communication and information; (2) unmet needs in follow-up care; (3) emotional impact and psychological support; (4) importance of patient and parental involvement; (5) desire for information and education; and (6) long-term concerns and support.

Clinicians, parents and patients expressed a need for tailored information resources to navigate FTP, particularly regarding timing. One parent shared, “*I don't know how to bring it up or talk with (my child) about it, how do I find out?*” (P1). The lack of tailored resources is perhaps unsurprising given that FTP is a relatively new technological advance, with research primarily focused on proving efficacy.^38^,^40^ Specialised pathways tailored to patients individual needs have been shown to help alleviate emotional burdens, provide timely interventions and prevent issues from escalating.^41 42^ A similar approach may have value for people with stored tissue.

There is global recognition of the need to align FTP follow-up care with existing oncofertility care models.^1143^,^45^ Young people have unique and rapidly evolving needs during their transition into young adulthood, making this a pivotal phase.^46^ Research should focus on creating survivorship care pathways that are both relevant and acceptable, encouraging timely support-seeking and improving decision-making. Tailored information resources such as specialised written or printed information or a dedicated online platform, which are integrated into a standardised pathway, may offer a way to optimise available resources, especially where care is constrained by budget or staffing limitations.

Our consultation highlighted unmet needs in follow-up care, particularly in ongoing information, education and psychosocial support. It provided the first insights into parents’ experiences after FTP, a previously unexplored area. These findings highlight critical gaps, particularly regarding the need for tailored resources to support parents navigating the FTP process. One clinician observed that while patients or parents are aware of the preservation process, they “*know nothing after that*” (P6). Existing research has documented inconsistencies in the provision of fertility preservation for young people with cancer in the UK,^4^ and our consultation revealed conflicting perspectives between oncologists and other specialists, leading to confusion and missed opportunities for coordinated care.

There is widespread support for more consistent and comprehensive survivorship care around fertility and reproductive health.^14 47 48^ Our findings affirm the need for research that addresses both medical outcomes and broader needs throughout the survivorship journey,^11 49 50^ aligning with national and international calls for improved survivorship care.^51^

Clinicians expressed a need for robust evidence on long-term outcomes to inform care after FTP and overcome the “*black hole of uncertainty” (P5*) around reproductive health after FTP. Longitudinal research could help track evolving needs, and a centralised database for FTP-related information would provide accessible, quality-assured data for clinicians. This would help harmonise knowledge across specialities and support informed clinical decision-making.

Patient-reported outcome measures have been shown to empower individuals to share their experiences and inform research priorities.^52 53^ Formal qualitative research methods may prove valuable in developing survivorship care pathways, resources and support services for high-quality, patient-centred care.

The development of patient-centred care following FTP must involve collaborative partnerships with service users to understand their unique perspectives. While the differing needs of clinicians, parents and young people align with previous research on long-term cancer survivorship,^54 55^ young people’s preference for sensitive conversations separate from their parents has been underexplored. This could indicate a need for individualised care pathways, possibly with separate pathways for parents and patients.

Participants in our consultation described the long-term emotional burden of isolation and anxiety associated with fertility concerns. As one young adult reflected, “*There wasn't any support […] it was just ‘get on with it yourself*…” (P2). Research has shown that while FTP can be a positive choice, it often brings fear, frustration and uncertainty.^19 20^ The extended interval between preservation in childhood and its later use in adulthood may amplify uncertainty, creating a greater burden compared with those undergoing FTP as adults.

There was a call for early, supportive education to minimise decision regret and assist with initiating discussions about reproductive health, particularly for teenagers who may view FTP as a ‘*backup plan’* (P4). Early education could help shift this perception, providing a clearer understanding of the process. The need for tailored emotional support was identified, with a clear demand for resources that assist in navigating reproductive care during survivorship.

Clinicians need more evidence-based data to identify key timepoints for information, clinical contact and referrals for psychological support. Given FTP’s sensitivity, clinicians need guidance on how to address it with confidence and care. Future research could focus on appropriate assessments for young adults, timing and support methods, particularly when managing difficult results or emotional responses.

Uncertainty around long-term care and emotional challenges in FTP survivorship were recurring themes. Parents described feeling unsupported in managing the emotional and logistical burdens. As one parent shared: “*I still feel like if I lose this bit of paper, I've only got that*.” (P1), emphasising the need for ongoing support between FTP and tissue use. Research should explore which professionals are best placed to provide this support, the format of these discussions and the duration of required assistance, to ensure comprehensive care that truly meets the needs of FTP survivors.

This study was limited by its small sample size and potential for selection bias. In the past two decades, only around UK 2000 children have stored ovarian or testicular tissue between the ages of 0–24 years, resulting in a limited pool of potential PPIE volunteers. Selective recruitment through advocacy and professional networks may have limited participation to those already engaged in PPIE activities, reducing the likelihood of including underrepresented groups. A larger, more diverse sample is required to validate and extend the findings.

To protect privacy, only minimal background data were collected, limiting contextualisation of participants’ responses. Informal interviews may have introduced recall bias, and limited rapport may have restricted the depth of discussion. Although this study yielded novel insights and a rich data set usually associated with formal qualitative research, the application of a PPIE framework rather than a formal qualitative methodology may limit its replicability.

### Conclusions

Findings from this PPIE consultation highlight critical gaps in follow-up care for cancer survivors with stored tissue, emphasising the need for tailored communication, emotional support and structured oncofertility care between FTP and tissue use. This consultation reinforces the complexity of FTP and its long-term implications, particularly the emotional and psychosocial challenges faced by families, and underscores the value of a standardised framework to guide clinicians and empower patients.

Application of a formal qualitative research method is necessary to generate results and insights that are relevant and generalisable to the population of young people with stored tissue. However, PPIE consultation has demonstrated the importance of involving both parents and young adults in designing care pathways to capture diverse insights and ensure real-world needs are addressed. Verbatim quotes from PPIE participants illustrated the genuine concerns and priorities for comprehensive follow-up care, encompassing informational, psychological and practical support.

Future research needs to develop interventions tailored to individuals who have undergone FTP.

## Supplementary material

10.1136/bmjopen-2024-088025 online supplemental file 1

## Data Availability

Data are available upon reasonable request.
