# Supplementary material for: Understanding the needs and experiences of young cancer patients, caregivers and healthcare professionals in the UK following childhood fertility tissue preservation (FTP): a qualitative study informed by patient and public involvement and engagement
Source: BMJ Open. 2025 Jul 28;15(7):e088025. doi: 10.1136/bmjopen-2024-088025 (PMC12306308; doi:10.1136/bmjopen-2024-088025)
Supplement: online supplemental file 1 [file bmjopen-15-7-s001.pdf]

Interview Guide: PPIE Interviews (Understanding the Needs and Experiences of Young Cancer Patients, Caregivers, and Healthcare Professionals After Childhood Fertility Tissue Preservation (FTP): Insights from Patient and Public Involvement and Engagement)

Goal: provide a flexible yet structured framework to ensure a safe and supportive environment for participants, allowing them to share their personal experiences in a c.

Given the sensitive nature of the topic, create a safe, respectful space for participants. Allow them to share their thoughts at their own pace and maintain flexibility in the interview process.

1. Interview Format

- Interviews to be conducted in an informal, one-to-one format, either via an online meeting platform (such as Zoom or Teams) or through telephone calls.
- Additional information or resources to be sent to participants via email, e.g. clarification of the purpose of the meeting and meeting links.
- Prior to the interview, informed consent is to be sought from all participants via email. Consent to be confirmed verbally at the beginning of each interview, ensuring that participants understand their role and how their data will be used.

2. Interview Process

a) Opening Question
Begin the interview with an open-ended question to allow participants to share their initial thoughts and experiences. Tailor the opening question depending on the participant's role:

- **For patients/parents**: "Can you tell me about any follow-up care you/your child has had after you had stored tissue?"
- **For clinicians**: "Can you tell me about the follow-up care you provide to patients after they have stored tissue?"

This question serves as a starting point to facilitate a natural and open conversation.

b) Probing Questions
Use probing questions to encourage participants to elaborate on specific aspects of their experience. For example:

- "What did you find helped you?"

- "What did you find less helpful?"
- "What do you think could help other people in your situation?"

The interviewer should adapt the questions to the flow of the conversation and ask the participant to elaborate on any new insights that emerge.

3. Data Collection

- During the interview, take notes to capture key points, significant quotes, and observations.
- After the interview, transcribe the notes, ensuring that verbatim quotes are included, and any meaningful interactions are accurately recorded.

4. Closing the Interview

- As the interview concludes, invite the participant to share any final thoughts or comments they may have on the topic.
- Thank the participants for their time and contribution.
- Provide participants with contact details in case they have follow-up questions or wish to withdraw their data after the interview.
