# Supplementary material for: Understanding the needs and experiences of young cancer patients, caregivers and healthcare professionals in the UK following childhood fertility tissue preservation (FTP): a qualitative study informed by patient and public involvement and engagement
Source: BMJ Open. 2025 Jul 28;15(7):e088025. doi: 10.1136/bmjopen-2024-088025 (PMC12306308; doi:10.1136/bmjopen-2024-088025)
Supplement: Supplementary file 2 [file bmjopen-2024-088025.reviewer_comments.pdf]

PEER REVIEW HISTORY

BMJ Open publishes all reviews undertaken for accepted manuscripts. Reviewers are asked to complete a checklist review form and are provided with free text boxes to elaborate on their assessment. These free text comments are reproduced below.

ARTICLE DETAILS

Title (Provisional)

Understanding the Needs and Experiences of Young Cancer Patients, Caregivers, and Healthcare Professionals After Childhood Fertility Tissue Preservation (FTP): Insights from Patient and Public Involvement and Engagement.

Authors

Mottram, Rebecca; Feltbower, Richard; Jones, Georgina Louise; Gelcich, Sarah; McLean, Katherine; Kelly, Catherine; Glaser, Adam

---

VERSION 1 - REVIEW

**Reviewer**	**1**
**Name**	**Moussaoui, Dehlia**
**Affiliation**	**Hôpitaux Universitaires Genève Département de médecine**
**Date**	**18-May-2024**
**COI**	**None.**

Thank you for the opportunity to review this manuscript, which is interesting and important. Nevertheless, there are a lot of underlying concerns that need to be addressed before considering publication.

Introduction:

- The introduction section is too long. In particular, the paragraph starting by "Given cancer's complexity..." on page 8 lacks focus and should be greatly shortened.

- The definition of "needs" and "unmeet needs" at the end of the same page are unnecessary.

- Many references do not seem to match the respective statement (e.g. reference 8, 9, 10, etc.) Authors should check every reference throughout the whole manuscript, and make sure they are the most appropriate.

Methods:

- This section is insufficiently detailed. In particular, participant recruitment is insufficiently developed. How was the study advertised? Was there a specific advertisement for each category of participants (parents, survivors, healthcare professionals)?

- Why did the authors choose to recruit different categories of participants, especially with such limited numbers of participants? Why did they not specifically focus on parents, survivors or healthcare professionals?

- When was the study performed? How long did the recruitment last?

- Was there any financial compensation offered to participants?

- Were the interviews audio recorded?

- Who did the interviews?

- Who did the transcription and the coding?

- Was there any software used for transcription and coding?

- How long did the interviews last approximately?

- The paragraph starting with "The participant cohort consisted..." on p.10 should be part of the results section and not methods

Results:

- Can the authors provide information about the participants (age, diagnosis, time since diagnosis, etc.)? This would help to interpret the results.

- Each citation should be referenced by whom said it (ex. clinician 1, parent 2, etc.)

Discussion:

- Same comment as above regarding references.

- The first two sentences of the discussion are unnecessary.

- The discussion should be better focus on discussing the main results of the study and how to guide future research and establish a standardised care pathway for patients (since these were the aims stated by the authors).

- Limitations should be discussed.

**Reviewer**	**2**
**Name**	**Albalushi, Halima**
**Affiliation**	**Sultan Qaboos University**
**Date**	**02-Jun-2024**
**COI**	**.**

This article addresses a fundamental issue in the field of cancer survivorship.

However, the number of participants is very low in all categories including parents (2), patients (1) and clinicians (4) with different specialities.

This number is insufficient to have a conclusion although the findings are not new and are the same as those published and known in the field.

I appreciate the work done by the team. Still, I think this work needs further expansion or if published to be published as short communication which also requires further expansion and a bigger sample size to be fit for publication.

**Reviewer**	**3**
**Name**	**Howard, A. Fuchsia**
**Affiliation**	**University of British Columbia, School of Nursing**
**Date**	**03-Oct-2024**
**COI**	**None**

This is an interesting study in an area that has yet to receive adequate attention and one that is clinically relevant. Overall, the manuscript provides a strong rationale for the research. Areas that would benefit from revision are as follows:

1. Study aim 2 (page 9): The second study aim (to guide future research to establish a care pathway) is not an aim of this particular study. While this study might inform the future development of further research and the development of a care pathway, this is not what was reported in the findings of this manuscript. I suggest reframing this so that it is not a study aim but rather part of the broader rationale for doing the research.

2. Methods: The authors indicate in the abstract that the design was patient and public involvement qualitative design using participatory methods. This contrasts with what is described in the methods section, where there is no mention or description of patient or public involvement as research team members. Furthermore, what is described in the methods section does not appear to be participatory methods, which would indicate the conceptualizing and carrying out of research in partnership with community members as team members, not necessarily study participants. It seems as though young people, patients and healthcare professionals were simply study participants – which does not constitute patient and public involvement or participatory methods.

3. Methods: I suggest authors consider including references to the methodological traditions or specific data collection and analysis techniques they drew upon.

4. Findings: The table of quotes does not add to the findings because the quotes are read out of context of the author’s narrative text. The author’s narrative text is essential to draw attention to and explain how participant quotes provide evidence for the authors' reporting.

With the quotes in the table, there is no guidance for how the reader is meant to interpret the string of quotes. I suggest deleting this table.

5. Findings: The wording used to report on the qualitative findings is somewhat inconsistent with the reporting of qualitative research in many places. Many statements read as facts rather than descriptions of data from participants that are evidence for the authors' points.

• For example, (Page 16) “Both parents and clinicians wanted guidance and resources….” These types of statements could be revised to something like, “The comments of both parents and clinicians suggested that they wanted guidance…”

• For example, (Page 17) “Long-term follow-up appointments may present and opportunity…” sounds like a statement that would be in the discussion. These types of statements could be revised to something like, “Clinicians often indicated that long-term follow-up appointments could potentially present an opportunity…”

6. Findings: While the abundance of quotes provides a solid foundation for the findings, the authors somewhat overused them without narrative text indicating what to pay attention to in the quotes. Additional textual descriptions accompanying the quotes would increase the readability of the findings.

7. Discussion: It is unclear how the first few paragraphs of the discussion relate to the study findings.

8. Discussion: There is no comparison of the study findings to other literature, which is necessary.

9. Study limitations: Other than the one bullet point (Page 7), there is no mention of study limitations in the discussion section. I suggest this be included.

VERSION 1 - AUTHOR RESPONSE

Reviewer	Reviewer comments	Author response	Changes made	Page No tracked paper
1	Introduction:<br>- The introduction section is too long. In particular, the paragraph starting by "Given cancer's complexity..." on page 8 lacks focus and should be greatly shortened.<br>- The definition of "needs" and "unmet needs" at the end of the same page are unnecessary.<br>- Many references do not seem to match the respective statement (e.g. reference 8, 9, 10, etc.) Authors should check every reference throughout the whole manuscript, and make sure they are the most appropriate.	We agree this can be made more concise.<br><br>We agree this can be removed.<br><br>There were some errors in the referencing due to a technical issue	The introduction has been shortened as advised.<br><br>This has been removed.<br><br>References have been checked and reformatted.	10<br><br>10<br><br>54

		with the citation software.		
1	Methods:<br>- This section is insufficiently detailed. In particular, participant recruitment is insufficiently developed. How was the study advertised? Was there a specific advertisement for each category of participants (parents, survivors, healthcare professionals)?	We agree the description of the method could have been clearer.	The methods section has been reworded to provide additional information.	14
1	Study aim 2 (page 9): The second study aim (to guide future research to establish a care pathway) is not an aim of this particular study. While this study might inform the future development of further research and the development of a care pathway, this is not what was reported in the findings of this manuscript. I suggest reframing this so that it is not a study aim but rather part of the broader rationale for doing the research.	We agree the aims were insufficiently clear.	The aims of the study have been clarified both in the abstract and on page 13.	4,13
1	Methods: The authors indicate in the abstract that the design was patient and public involvement qualitative design using participatory methods. This contrasts with what is described in the methods section, where there is no mention or description of patient or public involvement as research team members. Furthermore, what is described in the methods section does not appear to be participatory methods, which would indicate the conceptualizing and carrying out of research in partnership with community members as team members, not necessarily study participants. It seems as though young people, patients and healthcare professionals were simply study participants – which does not constitute patient and public involvement or participatory methods.	We agree that the papers focus on reporting the application of PPIE as a way to identify priority areas for research was not sufficiently clear. This paper reports on insights gained through the consultation phase of the project, which aimed to identify priority areas for future research to support the development of a tailored survivorship care pathway for childhood cancer survivors who have preserved tissue for future fertility. During this phase, PPIE participants	The abstract has been rewritten to clarify the focus of the paper<br><br>The method section has been reworded to provide additional detail on the application of a PPIE consultancy approach.	4, 14<br><br>14

		were engaged to share their views, experiences, and preferences on the topic, helping to inform the research focus. However, participants were not involved in the design of the interview guide or the methodology, as recruitment occurred after the study had been designed.		
2	Methods: I suggest authors consider including references to the methodological traditions or specific data collection and analysis techniques they drew upon.	We acknowledge that the focus of this paper was unclear in the first submission. The primary focus is on PPIE rather than the application of traditional research methodologies. PPIE does not generate data in the same way as conventional research approaches.	The methods section has been reworded to clarify the focus and methods of the paper and the overall focus of the paper has been clarified throughout.	14
2	Findings: The table of quotes does not add to the findings because the quotes are read out of context of the author's narrative text. The author's narrative text is essential to draw attention to and explain how participant quotes provide evidence for the authors' reporting. With the quotes in the table, there is no guidance for how the reader is meant to interpret the string of quotes. I suggest deleting this table.	We appreciate the reviewer's suggestion and agree this section could be stronger. We respectfully draw the reviewer's attention to consider the perspectives shared by participants as meaningful in themselves, and the importance of	We have redrafted the results/findings section and the table has been deleted. We have added narrative to explain the significance of the quotes and guide the reader as suggested. Please note that to fully address reviewers' comments, the length of the manuscript has significantly expanded the manuscript.	17

		acknowledging and giving weight to their voices.		
2	Why did the authors choose to recruit different categories of participants, especially with such limited numbers of participants? Why did they not specifically focus on parents, survivors or healthcare professionals?	We agree this needed clarification.<br>We assert that the smaller sample size is justified within the context of PPIE research, which emphasises qualitative insights over quantitative measures.<br><br>Engaging a variety of perspectives allowed for a more comprehensive understanding of the needs and experiences related to fertility tissue preservation. At this stage of the work we felt it was important not to make assumptions about who was most important to include, and by incorporating the range of participants, the insights reflect the complexities of FTP and its implications as well as enhancing the applicability of the findings.	We have taken care to clarify the scope and methods of the project throughout the paper.	
2	When was the study performed? How long did the recruitment last?<br>- Was there any financial compensation offered to participants?<br>- Were the interviews audio recorded?<br>- Who did the interviews?<br>- Who did the transcription and the coding?<br>- Was there any software used for	We agree this needed clarification.	Further detail has been added into the method section to address these omissions.	14

	transcription and coding?<br>- How long did the interviews last approximately?			
2	The paragraph starting with "The participant cohort consisted..." on p.10 should be part of the results section and not methods	We agree this would be better situated in the results section.	This detail has been moved to the results section.	18
2	Results:<br>- Can the authors provide information about the participants (age, diagnosis, time since diagnosis, etc.)? This would help to interpret the results.	We agree this information was missing.	Where detail is available, it has been added to the methods section to describe the participants.	18
2	- Each citation should be referenced by whom said it (ex. clinician 1, parent 2, etc.)	Noted.	Amended as requested.	18
2	Discussion:<br>- Same comment as above regarding references.<br>- The first two sentences of the discussion are unnecessary.<br>- The discussion should be better focus on discussing the main results of the study and how to guide future research and establish a standardised care pathway for patients (since these were the aims stated by the authors).<br><br>- Limitations should be discussed.	Noted.	We have redrafted the discussion to address these helpful comments and added in a section on the limitations.	43
3	This article addresses a fundamental issue in the field of cancer survivorship. However, the number of participants is very low in all categories including parents (2), patients (1) and clinicians (4) with different specialities.<br><br>This number is insufficient to have a conclusion although the findings are not new and are the same as those published and known in the field.<br>I appreciate the work done by the team. Still, I think this work needs further expansion or if published to be published as short communication which also requires further expansion and a bigger sample size to be fit for publication.	We agree that this is a small sample, but we respectfully draw the reviewer's attention to this as a traditional approach to gather detailed and nuanced data on lived experiences which is often difficult to achieve with a larger sample. The focus of the study was qualitative in nature, which is inherently more	Additional detail addressing these comments has been added to the method section to clarify the aims and design of this work.<br><br>We have also added in numbers from the focus group which was previously not submitted, and this has increased the number of participants as well as expansion of the work.	

		exploratory and in-depth. This depth of understanding was crucial to the aim of developing priority areas for novel research and the insights can be used to guide future, larger-scale research projects.<br><br>This research is one of the first to report on PPIE consultation in the context of FTP after childhood cancer and the inclusion of a diverse range of perspectives offers a new approach to understanding the gaps in care pathways. The findings offer specific, actionable insights that can influence practice in this emerging area.		
3	Findings: The wording used to report on the qualitative findings is somewhat inconsistent with the reporting of qualitative research in many places.	This paper reports on PPIE consultation work and does not use a traditional qualitative research design. Therefore, the expectations for sample size, methodological rigour, and reporting standards are not equivalent to formal qualitative research. However, we agree that this was not clearly	We have edited the focus of the paper to clarify that the purpose was to collaborate with relevant stakeholders to shape the research priorities and directions for future studies, rather than to generate definitive research data or novel findings.	

		defined in the previous draft.		
3	Findings:<br>Many statements read as facts rather than descriptions of data from participants that are evidence for the authors' points.<br>• For example, (Page 16) “Both parents and clinicians wanted guidance and resources….” These types of statements could be revised to something like, “The comments of both parents and clinicians suggested that they wanted guidance…”<br>• For example, (Page 17) “Long-term follow-up appointments may present and opportunity…” sounds like a statement that would be in the discussion. These types of statements could be revised to something like, “Clinicians often indicated that long-term follow-up appointments could potentially present an opportunity…	We agree this could have been clearer.	To address these concerns we have revised the wording in the findings section to more accurately reflect participant perspectives. Rather than stating findings as definitive facts, we have attributed the comments to the data, e.g. "The comments of both parents and clinicians suggested..." and "Clinicians often indicated..." We hope that this maintains the integrity of the process and clearly expresses findings as shaped by participants input.	17
3	Findings: While the abundance of quotes provides a solid foundation for the findings, the authors somewhat overused them without narrative text indicating what to pay attention to in the quotes. Additional textual descriptions accompanying the quotes would increase the readability of the findings.	We agree narrative was missing.	Narrative text has been added before and after the quote to explain the significance of the participant's response and to help guide the reader’s attention to the key themes emerging from the quotes.	17
3	Discussion: It is unclear how the first few paragraphs of the discussion relate to the study findings.	Noted.	The discussion section has been significantly edited to address these comments, including a revision of the first few paragraphs.	45
3	Discussion: There is no comparison of the study findings to other literature, which is necessary.	Noted.	The discussion section has been edited to address these comments, including a revision of the first few paragraphs.	45

3	Study limitations: Other than the one bullet point (Page 7), there is no mention of study limitations in the discussion section. I suggest this be included.	Noted.	We have added a section on the limitations.	50

VERSION 2 - REVIEW

**Reviewer**	**1**
**Name**	**Moussaoui, Dehlia**
**Affiliation**	**Hôpitaux Universitaires Genève Département de médecine**
**Date**	**03-Feb-2025**
**COI**	

I thank the authors for their revised manuscript, which provides much clearer information.

I think they have answered appropriately to most comments.

I have two last questions/comments:

Results page 5, line 31-39 (page and line number refers to the tracked changes document): the sentence about participants number is unclear as it is stated that "Six adult participants took part in a focus group... three who were cancer childhood survivors... and two who were parents...." Who is the 6th participant?

Discussion - a discussion of the limitations is usually needed in the discussion section and not only as bullet points. The authors say that they have added a section (page 50 of the tracked changes document?? I may not have the same page numbering) but I can't see it.

VERSION 2 - AUTHOR RESPONSE

**Reviewer**	**Reviewer comments**	**Author response**	**Changes made**	**Page No tracked paper**
Reviewer: 1	Results page 5, line 31-39 (page and line number refers to the tracked changes document): the sentence about participants number is	We acknowledge this was not clear and have revised this line.	Twelve unique participants contributed to the study. Six adult participants took part in a focus group, three who were childhood cancer survivors with stored fertility tissue, two who	

	unclear as it is stated that "Six adult participants took part in a focus group... three who were cancer childhood survivors... and two who were parents...." Who is the 6th participant?		were parents of pre-adolescent children who had stored ovarian or testicular tissue in early childhood and one who was a clinician.	
Reviewer: 1	Discussion - a discussion of the limitations is usually needed in the discussion section and not only as bullet points. The authors say that they have added a section (page 50 of the tracked changes document?? I may not have the same page numbering) but I can't see it.	Formatting had prevented the previously written limitations section added to the discussion from appearing. This has been put back in at the end of the discussion. Please accept our apologies.	This study was limited by its small sample size and potential for selection bias. In the past two decades, only around 2000 UK children have stored ovarian or testicular tissue between the ages of 0-24 years, resulting in a limited pool of potential PPIE volunteers. Selective recruitment through advocacy and professional networks may have limited participation to those already engaged in PPIE activities, reducing the likelihood of including underrepresented groups. A larger, more diverse sample is required to validate and extend the findings.<br><br>To protect privacy, only minimal background data were collected, limiting contextualisation of participants' responses. Informal interviews may have introduced recall bias, and limited rapport may have restricted the depth of discussion.	

			Although this study yielded novel insights and a rich data usually associated with formal qualitative research, the application of a PPIE framework rather than a formal qualitative methodology may limit its replicability.	
